# A Pain in the Butt: The Association Between Endo-Parasite Diversity and Horn Growth in Rocky Mountain Bighorn Sheep

**DOI:** 10.3390/pathogens14070623

**Published:** 2025-06-23

**Authors:** Tanisha C. Henry, Samridhi Rijal, Joana Alves, Peter Neuhaus, Susan Kutz, Kathreen E. Ruckstuhl

**Affiliations:** 1 Department of Biological Sciences, University of Calgary, Calgary, AB T2N 1N4, Canada; tanisha.henry@ucalgary.ca (T.C.H.); samridhir@gmail.com (S.R.); pneuhaus@ucalgary.ca (P.N.); 2 TERRA Associate Laboratory, Department of Life Sciences, Centre for Functional Ecology (CFE), University of Coimbra, 3000-456 Coimbra, Portugal; jalves@uc.pt; 3 Faculty of Veterinary Medicine, University of Calgary, Calgary, AB T2N 4Z6, Canada; skutz@ucalgary.ca

**Keywords:** horn growth, age, lungworms, nematodes, *Eimeria*, parasite diversity, bighorn sheep

## Abstract

(1) *Background*: Parasites can significantly impact growth, reproductive success, and the development of secondary sexual characteristics in various species. Our study investigated the effect of gastrointestinal and lungworm parasite infections on the total annual horn growth of male bighorn sheep (*Ovis canadensis canadensis*) in Sheep River Provincial Park, Alberta, Canada. (2) *Methods*: We collected fecal samples of individually marked male bighorns over two years to investigate parasite egg and oocyst numbers and parasite diversity in feces, and how these could potentially affect their annual horn growth. (3) *Results*: We found that parasite species richness, year, age class, and the interaction between age class and species richness were significant predictors of horn growth, while individual parasite species did not have a significant effect. Notably, parasite species richness positively affected horn growth in young males, whereas it did not predict horn growth in adult and old males. (4) *Conclusions*: One possible explanation is that young males might prioritize resources for body and horn growth, potentially at the expense of immunity or parasite resistance. Our finding contradicts the idea of parasite-mediated sexual selection, where traits like bright plumage signal good health and parasite resistance.

## 1. Introduction

The development of elaborate male ornamentation and weaponry, such as colourful plumage, horns, and antlers, is driven by intense sexual selection, specifically through male–male competition and female mate choice [1]. These secondary sexually selected traits often confer fitness benefits in the form of increased reproductive success, but are costly to develop and maintain [2,3,4]. Consequently, these traits are considered honest signals of an individual’s quality to potential mates, as only high-quality individuals can afford the associated energetic costs.

In many species, males are more vulnerable to parasites, which have been linked to high testosterone levels [5,6]. Several studies have identified a negative relationship between both ecto- and endo-parasite infections and the growth of secondary sexual characteristics. For instance, male and female Cape bison (*Syncerus caffer*) with increased parasite richness exhibited significantly reduced horn growth [7]. Similarly, house finches (*Carpodacus mexicanus*) with higher parasite loads had diminished secondary sexual trait development [8]. Due to the cumulative and competitive effects of coinfection, it is critical to investigate both parasite species richness and the infection intensity of individual parasite species [9].

The immunocompetence-handicap hypothesis [5] proposes a trade-off between immune function and the growth of secondary sexual traits. Elevated levels of testosterone can stimulate mating behaviors (e.g., vocalization, increased agonistic interactions, mating dances) and the growth of secondary sexual traits. However, testosterone is an immunosuppressant, and elevated levels can lead to increased susceptibility to disease and parasitic infections across vertebrate taxa [10]. For example, because of increased testosterone levels, intact male reindeer (*Rangifer tarandus*) suffered more intense infections of warble fly larvae than castrated males and females, who had similar infection intensities [11]. Furthermore, old elk (*Cervus elaphus canadensis*) with large antlers are more likely to be infected with scabies post-rut, as dominant males seem to invest more in antler growth and reproduction [12], likely at the expense of immune function. Lacertid lizards (*Psammodromus algirus*) treated with testosterone develop brighter nuptial colouring, but also suffer larger infestations of ticks and increased disappearance rates (presumed dead) [13], further suggesting a trade-off between reproduction and immune function/survival facilitated by testosterone’s immunosuppressive effects.

Previous research has shown that sustained hunting [14,15], environmental pressures and demography [16], and disease (e.g., “Lumpy Jaw”, [17]; pneumonia, [18]) can reduce horn size in bighorn sheep (*Ovis canadensis canadensis*). However, the effect of parasite infection on bighorn sheep horn growth remains unstudied. Parasite infections are often multilayered [19], with multiple species infecting a host simultaneously, potentially compounding their impact on horn growth.

Bighorn sheep in our study area have been found to carry various gastrointestinal parasites, including Strongyles (including *Teladorsagia* spp., *Ostertagia* spp., *Trichostrongylus* spp., and *Cooperia oncophora*), *Nematodirus* spp., *Marshallagia marshalli*, *Eimeria* spp., *Moniezia* spp., and lungworms (*Protostrongylus* spp.) [20]. These parasites have different health effects depending on the severity of infection. For example, Strongyles can cause diarrhea, weight loss, and anemia. *Marshallagia marshalli* can damage the abomasum, leading to reduced nutrient absorption and poor health. *Eimeria*, a coccidian parasite, can cause coccidiosis, associated with diarrhea, dehydration, and weight loss. *Moniezia*, a tapeworm, can reduce feeding efficiency and cause weight loss, while lungworms can cause respiratory issues, such as bronchial irritation and coughing, likely affecting oxygen saturation in the blood [20].

Given the paucity of information on the effect of parasite infections on the growth of secondary sexual traits in ungulates, specifically bighorn sheep, we aim to address the following question: how do gastrointestinal and respiratory parasite infections impact the total annual horn growth of Rocky Mountain bighorn sheep males? We hypothesize that more heavily parasitized individuals will exhibit reduced total annual horn growth, as energy is allocated to immune function rather than secondary sexual trait growth. Furthermore, we predict that (1) more intense infections (higher fecal shedding of eggs) will lead to decreased annual horn growth and (2) higher parasite species richness will lead to decreased annual horn growth.

## 2. Materials and Methods

### 2.1. Study Site and Animals

This study was conducted in Sheep River Provincial Park (SRPP), Alberta, Canada (50°40′ N, 114°40′ W). The park is in the foothills of the Rocky Mountains and is characterized by open grassy meadows, coniferous and aspen forests, and the Sheep River Valley. Rocky Mountain bighorn sheep males grow large horns with annuli (much alike tree rings), facilitating reliable age determination and measurement of annual horn growth. The SRPP population of bighorn sheep is part of an ongoing study, with a long-term data set compiled by Dr. K.E. Ruckstuhl since 1994. The SRPP sheep are captured as lambs, during which time DNA is collected and they are fitted with standard Alflex ear tags. Each ear tag combination corresponds to an individual ID number, allowing us to track individuals throughout their lives [21]. Additionally, these sheep are habituated to our presence, allowing for reliable data collection.

### 2.2. Fecal Parasite Screening

Alberta bighorn sheep are commonly infected with seven endo-parasite groups [22] that can be quantified using fecal egg counts (FEC). FEC offers a non-invasive method of assessing parasite infection levels [23,24]. To collect fecal samples, individually ear-tagged bighorn sheep were observed while bedding or grazing, then locations of defecation were noted for each sheep, and used to confidently pair the fecal samples with individual sheep. Once the sheep had moved, feces were collected [20]. The samples were stored in separate vacuum-sealed plastic bags in a cooler and then transferred to a refrigerator [20].

The Modified Wisconsin Double Centrifugation method was used to extract fecal parasites, following the Standard Operating Procedure 3 from the Alberta CWHC Parasitology Lab (WCVM Parasitology diagnostic techniques handbook, 2002, see also https://wcvm.usask.ca/learnaboutparasites/diagnostics/quantitative-wisconsin-technique-wcvm-lab.php, accessed on 19 June 2025), with the modification of using 4 g of fecal sample and two layers of cheesecloth to strain the fecal solution. The egg/oocyst count data were divided by the weight of the fecal sample (4 g) to calculate the number of eggs/oocysts per gram of feces (EPG). After processing the samples, egg counts were conducted using a 10 × 10 ocular microscope, typically within 1 to 2 days of sample collection, but no later than 5 days after collection [20]. FEC were measured as the number of eggs per four grams of feces. Strongyle eggs could not be reliably differentiated to genus or species and were grouped together.

Over the study, 191 fecal samples were collected from 31 rams, as follows: 80 from young males, 86 from middle-aged males, and 25 from old males. Data on both parasite prevalence and diversity and horn growth were collected in 2016 and 2017 for 31 bighorn rams aged 1 to 13 years old. There were 18 young rams (from 1 to 4 years old), 16 adult rams (5 to 8 years old), and 3 old rams (9 years and older). Few rams live to reach old age due to trophy hunting and natural mortality, resulting in a small sample size for the old age class, which may limit statistical power, and is not under our control. Lambs were excluded from analysis, as their growth is influenced by maternal care. Although data were collected for *Trichuris* spp. in 2017 (not 2016), they were not included in the analysis, as there were only 8 non-zero values.

### 2.3. Horn Growth Data

All rams were of known age (ear-tagged as lambs or subadults), and annual horn growth was cross-referenced and measured for the years parasite data were collected (2016 and 2017). In cases where manual post-mortem or capture measurements were available, these measurements were used. If there were no manual measurements available (i.e., the individual is still alive or disappeared and is presumed dead), photographs were used to digitally measure annual horn growth (length between two annuli). Photographs used for digital analysis post-dated the collection of parasite data and were only used if they showed a full season of growth. Photographs taken in profile or head-on were used for analyses. Annual growth was measured for each horn of each ram in ImageJ 1.54g [25] using ear tag height (3.7 cm) as a reference scale [26]. To minimize inaccuracies in measurements stemming from inter-personal or inter-image differences, all images were analyzed by the same person (TCH), and 3 photos were analyzed per horn, per ram, after which the horn growth measurements were averaged.

### 2.4. Data Analysis

To evaluate the effects of gastrointestinal parasite burden and host age on horn growth in bighorn sheep, we used a linear mixed-effects model, with all predictor variables standardized. Total horn growth was modeled as the response variable, and fixed effects included age class (i.e., young = aged 2–4, adult = aged 5 to 8, and old = aged 9 to 14 years old); mean yearly parasite counts for Strongyles, *Nematodirus*, *Moniezia*, and *Eimeria* spp. (all z-transformed); parasite species richness; year; and the interaction between age class and parasite species richness. To account for repeated measurements within individuals between years, Sheep ID was included as a random factor.

The model was fitted using the lmer function from the lme4 package [27,28]. Standardized variables were used to improve model interpretability and numerical stability. Two observations with extreme outliers were removed based on exploratory data analysis. Model assumptions—including linearity, homoscedasticity, normality of residuals, and absence of influential points—were thoroughly evaluated using the performance package. No major violations were detected. Predictor multicollinearity was assessed using Variance Inflation Factors (VIF) and found to be within acceptable limits (<3).

We tested the significance of fixed effects using Type III ANOVA with Satterthwaite’s approximation for degrees of freedom, as implemented in the lmerTest package [3]. Pairwise comparisons and post hoc interaction analyses were performed using the emmeans package [29], with Tukey adjustment for multiple testing. All statistical analyses were conducted using R version 3.5.0 [30]. Statistical significance was considered at *p* < 0.05, and results are presented as estimated means ± standard error (SE), unless otherwise indicated.

## 3. Results

**Figure 1:**
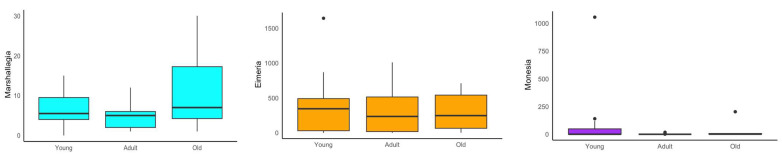

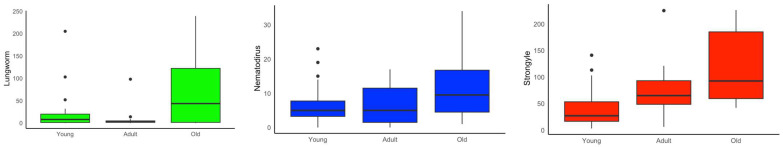
Counts of the different parasites found in bighorn sheep feces belonging to different age classes of males (young = aged 2–4, adult = aged 5 to 8, and old = aged 9 to 14 years old); these counts are numbers of eggs/larvae/oocysts per 4 g of wet fresh feces. Shown are median counts with interquartile (IQR) ranges (boxes), lines extending from the smallest and largest values within 1.5 times the IQR from the lower and upper quartiles, and dots representing potential outliers.

The median parasite intensity for the young, adult, and old age classes are presented in Figure 1.

None of the individual parasite intensities, which include Strongyles, *Nematodirus*, *Moniezia*, or *Eimeria*, were significantly associated with horn growth (*p* > 0.25 in all cases) (Table 1 and Table 2).

**Figure 2:**
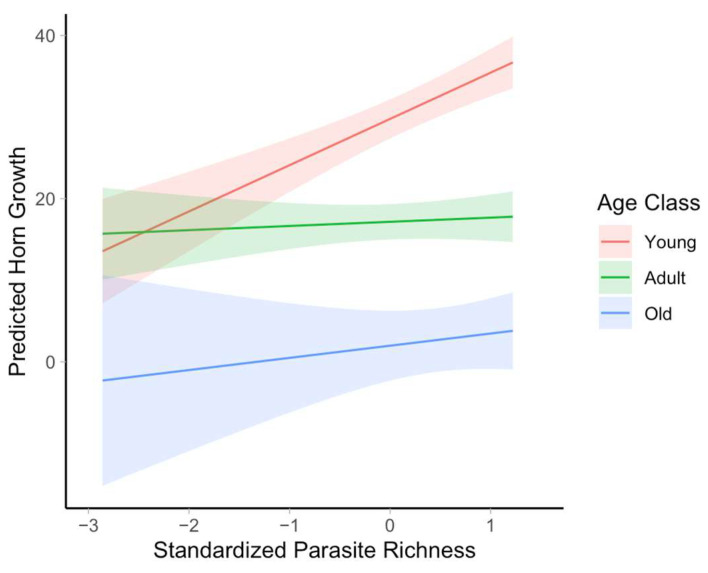
Predicted effects of parasite species richness on bighorn sheep horns growth across age classes, based on the linear mixed-effects model. Lines represent marginal predictions with 95% confidence intervals for the interaction between age class and standardized parasite species richness.

Model results revealed a strong effect of age class on horn growth (F_(2, 24)_ = 83.72, *p* < 0.001), with younger individuals (2–4 years old) having significantly higher horn growth (β = 12.61 ± 1.33 SE, *p* < 0.001) and older individuals (9–14 years old) having lower growth (β = –15.19 ± 2.32 SE, *p* < 0.001) comparatively to the adult (5–8 year old) age class (Table 1 and Table 2). Parasite species richness had a positive, though nonsignificant, association with horn growth in adult rams (β = 0.51 ± 0.93 SE, *p* = 0.586). However, a significant interaction between age class and parasite species richness (F_(2, 33)_ = 9.56, *p* < 0.001) indicates that this relationship between parasite species richness and horn growth varies by age. Specifically, a strong positive association between parasite species richness and horn growth was observed in young individuals (β = 5.16 ± 1.21 SE, *p* < 0.001). While no such effect was detected in adults or older individuals (Table 1 and Table 2, Figure 1). These results indicate that the effect of parasite species richness on horn growth is age-dependent, and unexpectedly positive in younger animals (Figure 2, Table 1 and Table 2).

Year had a moderate effect (F_(1, 24)_ = 6.48, *p* = 0.018), reflecting temporal variability in horn growth, with reduced horn growth observed in 2017 compared to 2016 (Table 1 and Table 2).

Finally, the model included Sheep ID as a random effect to account for repeated measures within individuals. The estimated variance attributable to individual identity was 5.13 (SD = 2.26). This indicates that approximately 34% of the variation in horn growth was due to consistent among-individual differences not explained by age, year, or parasite measures.

## 4. Discussion

Our study found that only parasite species richness, year, age class, and the interaction between age class and parasite species richness were significant predictors of the total annual horn growth in male bighorn sheep, while none of the individual parasite species had a significant effect. Notably, we observed a positive effect of parasite species richness on horn growth in young males, while parasite species richness did not predict annual horn growth in adult and old males. These results highlight that horn growth varies markedly by age class and is modulated by parasite species richness in young individuals, suggesting a potential age-specific tolerance or resource reallocation strategy.

This finding contradicts our initial hypothesis that parasites would negatively affect horn growth. One possible explanation is that young males might prioritize resources for body and horn growth, potentially at the expense of immunity or parasite resistance. This aligns with the idea that testosterone, which promotes the development of secondary sexual traits like horn growth, also suppresses the immune system [5]. However, this does not fully explain why only young rams with larger horn growth had higher parasite diversity. It is possible that young males are more susceptible to parasitic infections than adult males because their immune system is not yet fully developed, but this would require further exploration. Across mammalian species, the relationships between parasite infection and immunosuppressant hormones are variable and complex [31]; however, without experimental manipulation of parasite loads it remains challenging to differentiate between the cause and the consequences.

Our findings further contrast with those of [9], who reported that increased parasite species richness significantly reduced horn growth in both male and female Cape bison (*Cyncerus caffer*). An increased parasite species richness might indicate a higher overall susceptibility to infection, potentially related to age or the relative naivety to parasitic infections and immune responses of young animals.

In several species, including long-finned pilot whales (*Globicephala melas*; [32]), coral reef fishes [33], and wood frogs (*Lithobates sylvaticus*; [34]), a positive relationship between host age and parasite species richness has been documented. However, our study found the opposite. The youngest age class, which had the highest parasite species richness, also exhibited the largest horn growth. This finding contradicts [4]’s idea of parasite-mediated sexual selection, where traits like the bright plumage of male red jungle fowl (*Gallus gallus*) signal good health and parasite resistance.

Parasite infection intensity has been shown to influence life-history traits and behavior across species. For example, male red jungle fowl with larger combs and brighter plumage are preferred by females [35]. However, these males also have elevated testosterone levels and lower lymphocyte counts, suggesting increased disease susceptibility [35]. Furthermore, males with reduced parasite infection intensities typically have brighter plumage [4], indicating a complex relationship between honest signaling of quality, mate choice, and immune function.

In bighorn sheep, horn length, body size, and age correlate with dominance and reproductive success [36,37]. Previous studies on Dall’s sheep (*Ovis dalli*; [38]), Soay sheep (*Ovis aries*; [39]), and red deer (*Cervus elaphus*; [40]) have shown that an increased parasite burden can reduce body mass. This suggests that the relationship between parasite burden, horn size, body mass, and age can influence mating behaviour, reproductive success, and survival. Notably, our model revealed substantial among-individual variation in horn growth, with the random effect of Sheep ID accounting for approximately 34% of the total variance. This persistent individual heterogeneity likely reflects unmeasured factors such as genetics, maternal investment, or early-life conditions [41], or it could simply be a reflection that those males who eat more grow larger horns but also ingest more parasites. Such individual differences are increasingly recognized as central to understanding phenotypic performance and life-history strategies in wild populations [42], and may play a critical role in shaping horn development beyond age or parasite pressure.

Although our results showed a significant effect of year on horn growth, we do not believe this to be a notable finding. Due to the schedule of fecal sample collection, our study was limited to two years (2016 and 2017), with reduced horn growth observed in 2017 compared to 2016. Without a dataset spanning a larger length of time, it is impossible to confidently comment on the relationship between year, parasite intensity, and horn growth. There is a strong seasonal effect on parasite intensity [20], as well as temporal effects relating to weather, climactic events, and in extension, forage quality on horn growth in bighorn sheep [16]. It is plausible that these factors explain our observed effect of year on horn growth.

In summary, our expectation of a trade-off between horn growth and parasite infection, where parasite species richness would reduce horn growth, was not supported. This suggests that interactions between testosterone-induced immunosuppression, parasite biology (e.g., seasonality), host ecology (e.g., growth and mating seasons), and both intra- and intersexual selection are complex and require further research. Targeted experiments, such as infecting males with specific parasites or treating some with anthelmintic drugs, could reveal the potential direct impact of parasites on male horn growth and fitness, as our current study remains correlational.

## Figures and Tables

**Table 1:** Results of the Type III analysis of variance (Satterthwaite’s method) testing the effects of age class, parasite metrics (number of eggs, or oocysts per 4 g of feces), and year (2016 and 2017) on male bighorn sheep yearly horn growth (length). Significant results are bolded.

Effect	Sum of Squares	Mean Squares	df	F-Value	*p*-Value
Age class	1682.85	841.43	2	83.72	**<0.001**
Strongyle egg counts	Data2.84	2.84	1	0.28	0.593
*Nematodirus* egg counts	4.72	4.72	1	0.47	0.498
*Moniezia* egg counts	12.40	12.40	1	1.23	0.275
*Eimeria* oocyst counts	0.17	0.17	1	0.02	0.898
Parasite Species Richness	91.34	91.34	1	9.09	**0.005**
Year	65.11	65.11	1	6.48	**0.018**
Age class × Parasite Species Richness	192.17	96.08	2	9.56	**<0.001**

**Table 2:** Coefficient estimates from the linear mixed-effects model assessing the influence of age class, parasite counts (standardized), parasite species richness, and year on total horn growth. Estimates are presented with standard errors (SE), *t* test statistics, degrees of freedom (df), *p*-values, and 95% confidence intervals. Significant effects are bolded.

Predictor	Estimate	SE	*t* Test	df	*p*-Value	95% CI Lower	95% CI Upper
(Intercept)	20.047	1.294	15.497	36.01	**<0.001**	17.42	22.67
Age class—Old	−15.185	2.317	−6.554	17.83	**<0.001**	−20.06	−10.31
Age class—Young	12.612	1.330	9.480	37.92	**<0.001**	9.92	15.30
Strongyle	−0.392	0.737	−0.531	37.47	0.598	−1.89	1.10
*Nematodirus*	0.450	0.657	0.686	31.89	0.498	−0.89	1.79
*Moniezia*	−3.092	2.784	−1.111	31.53	0.275	−8.77	2.58
*Eimeria*	−0.095	0.731	−0.130	36.68	0.897	−1.58	1.39
Parasite species richness	0.511	0.928	0.550	32.83	0.586	−1.38	2.40
Year—2017	−3.722	1.462	−2.545	24.14	**0.018**	−6.74	−0.71
Age class—Old × Parasite species richness	0.981	2.051	0.478	30.06	0.636	−3.21	5.17
Age class—Young × Parasite species richness	5.159	1.210	4.265	37.15	**<0.0001**	2.71	7.61

## Data Availability

Data and R code (R version 3.5.0) will be publicly available upon acceptance of the article for publication in MDPI *pathogens*.
